# Ionic thermoelectrics goes epidermal theranostics

**DOI:** 10.1093/nsr/nwag198

**Published:** 2026-03-30

**Authors:** Jing Liu, Yongyuan Kang, Peipei Liu, Qinglin Jiang, Fengxing Jiang, Changyou Gao, Yuguang Ma

**Affiliations:** Jiangxi Provincial Key Laboratory of Flexible Electronics, Jiangxi Science and Technology Normal University, China; MOE Key Laboratory of Macromolecular Synthesis and Functionalization, Department of Polymer Science and Engineering, Zhejiang University, China; Jiangxi Provincial Key Laboratory of Flexible Electronics, Jiangxi Science and Technology Normal University, China; Guangdong Basic Research Center of Excellence for Energy and Information Polymer Materials, Guangdong Provincial Key Laboratory of Luminescence From Molecular Aggregates, State Key Laboratory of Luminescent Materials and Devices, Institute of Polymer Optoelectronic Materials and Devices, South China University of Technology, China; Jiangxi Provincial Key Laboratory of Flexible Electronics, Jiangxi Science and Technology Normal University, China; MOE Key Laboratory of Macromolecular Synthesis and Functionalization, Department of Polymer Science and Engineering, Zhejiang University, China; Guangdong Basic Research Center of Excellence for Energy and Information Polymer Materials, Guangdong Provincial Key Laboratory of Luminescence From Molecular Aggregates, State Key Laboratory of Luminescent Materials and Devices, Institute of Polymer Optoelectronic Materials and Devices, South China University of Technology, China

## Abstract

Highlighting a major paradigm shift, this perspective envisions the novel use of ionic thermoelectrics as smart epidermal theranostics, unlocking new bioelectric strategies for advanced wound management and tissue regeneration.

Smart epidermal theranostics requires continuous and autonomous power supply. Among the various available energy sources, body heat is particularly attractive due to its stability, ubiquity, and the absence of external energy input. Ionic thermoelectrics (i-TE), operating via thermodiffusion-driven Soret effects and temperature-gradient-dependent redox reactions (thermogalvanic effects) [1], can directly convert the small skin-level temperature gradients, typically only 1–5 K, into millivolt-level electrical signals [2,3]. This capability establishes i-TE as a viable route toward self-powered epidermal theranostics. As comprehensively illustrated in Fig. 1, i-TE represents a direct interface between biological systems and electronics. To realize its full clinical potential, however, a paradigm shift is required. Currently, i-TE devices rely on *ex situ* fabrication and static encapsulation, often encountering conformal mismatches and chronic inflammatory rejection. Future designs must pivot toward *in situ* deployment via self-organized spraying for seamless integration coupled with dynamic self-sacrificing encapsulation to actively quench reactive oxygen species. By synergizing these engineering materials with core thermogalvanic and thermodiffusion mechanisms, next generation i-TE platforms can concurrently deliver precise diagnostic monitoring and targeted therapeutic bioelectric cues for high-quality regenerative repair.

**Figure 1.**
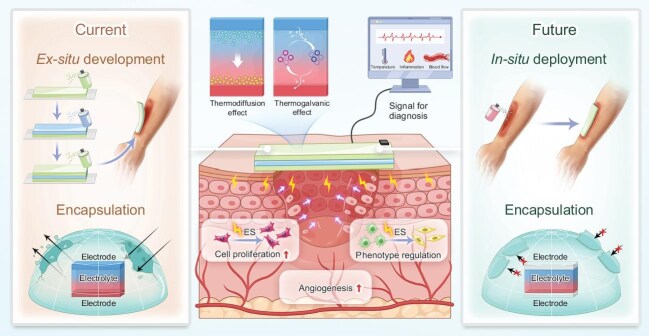
Concept and paradigm shift of ionic thermoelectric epidermal electronics. Beyond converting body heat into diagnostic signals and therapeutic electrotherapy, future development must transition from current *ex-situ* fabrication and static encapsulation to *in-situ* self-organized deployment and dynamic self-sacrificing encapsulation.

In epidermal electronics, wound management remains challenging. Traditional treatments overlook endogenous bioelectrical regulation, such as the transepithelial potential (TEP) that guides cell migration upon injury. Exogenous electrical stimulation (ES) effectively mimics this wound electric field to promote regenerative repair [4]. Furthermore, abnormal scar formation driven by overactivated fibroblasts remains a major adverse outcome. Applying stable ES significantly suppresses pro-fibrotic signaling and fibroblast-to-myofibroblast trans-differentiation, thereby enabling high-quality, scarless healing [4,5].

The i-TE epidermal electronics serve dual therapeutic and diagnostic functions. Therapeutically, a recent thermogalvanic hydrogel utilizing the Fe^2+^/Fe^3+^ redox pair directly transduces 1–3 K skin temperature differences into millivolt-level potentials [6]. This self-sustained ES supplements the endogenous wound electric field to promote cell migration, while Fenton-reaction-generated radicals provide synergistic antibacterial effects. In diagnostics, Soret-effect-based flexible ionogels directly convert thermal and mechanical stimuli into electrical signals for battery-free physiological sensing [7]. Additionally, i-TE can function as self-powered gates for organic electrochemical transistors, amplifying subtle thermal fluctuations into processable outputs [8].

While i-TE shows growing promise for self-powered theranostics, their further development toward practical and eventually clinical applications requires resolving several intertwined challenges. For near-term translation, overcoming materials and system-level constraints regarding biocompatibility, long-term stability and conformal integration is an urgent priority to ensure device safety and controllability. Concurrently, while elucidating the cellular-scale mechanism of action remains a medium- to long-term scientific opportunity, it is an indispensable guidepost. Establishing a causal link between material operational modes and biological outcomes provides fundamental design parameters for next-generation polymers. Although Soret and thermogalvanic effects are fundamentally distinct in microscopic thermodynamic origins (steady-state ionic gradient versus dynamic Faradaic current), they are practically unified within the epidermal theranostics framework. Both macroscopically transduce subtle skin temperature gradients into physiologically meaningful electrical cues, yet how these specific signals differentially activate downstream biological pathways remains an open question.

Concurrently, the pursuit of higher thermopower must be reconciled with the imperatives of biocompatibility and long-term stability. High-concentration electrolytes and volatile solvents raise significant concerns regarding long-term ion and solvent leakage, cytotoxicity, and chronic immune responses. Beyond developing intrinsically biocompatible and redox stable i-TE materials to minimize polymer degradation and adverse biological interactions, robust encapsulation strategies are necessitated. Moving beyond static barriers, dynamic self-sacrificing encapsulation [9] resolves the spatiotemporal contradiction of reactive oxygen species (ROS) regulation. While transient radicals provide early-stage antibacterial action, clearing excess ROS during proliferation is a prerequisite for scarless healing. A sacrificial layer degrading in the inflammatory microenvironment actively quenches late-stage oxidative stress. This trade-off between encapsulation degradation and long-term device stability represents a necessary paradigm shift. Since wound treatment cycles are short-term, prioritizing dynamic microenvironment regulation over permanent structural integrity ensures safe, high-quality repair while maintaining bioelectrical transmission. To ensure that the voltage consistently surpasses the effective biological threshold (e.g. 10–60 mV) amidst environmental thermal fluctuations, innovative structural designs are imperative. Configuring i-TE units into series-connected arrays effectively multiplies output voltage. Additionally, incorporating asymmetric heat dissipation and thermal insulation within the encapsulation actively shields the device from thermal interference, stably maintaining the cross-device temperature gradient in open settings.

Finally, addressing manufacturability and integration with existing theranostics platforms requires a manufacturing paradigm shift. Conventional top-down integration is ill suited for creating the large area conformal interfaces required for practical clinical applications. Instead, a transformative ‘bottom-up’ self-assembly alternative has emerged. For instance, one-step blend spraying of immiscible polymer solutions triggers spontaneous macroscopic phase separation, forming freestanding multilayer structures with spatially segregated functions [10]. Extending this ‘manufacturing-by-self-organization’ principle to i-TE, a multicomponent ink could spontaneously segregate upon spraying to form a continuous, three-dimensional functional structure (e.g. electrodes sandwiching an electrolyte). This directly yields a ready-to-use device, bypassing individual component alignment and fundamentally addressing integration bottlenecks. However, applying this self-assembled spraying technology to human epidermis requires overcoming challenges from nonplanar topologies and dynamic stretching. Future research must optimize the rheological properties of multicomponent inks to ensure structural uniformity on complex topographies. Furthermore, strengthening interlayer cohesion and tissue−interface adhesion, alongside elastic modulus matching with skin, ensures mechanical stability during vigorous movement. Crucially, by prioritizing the generation of functional bioelectrical signals over traditional high-power conversion efficiency, we call for the establishment of a novel evaluation paradigm tailored for epidermal ionic thermoelectrics. Future assessments should shift core criteria toward metrics with greater clinical relevance, such as effective ion flux per unit temperature difference, interfacial charge transfer efficiency and dynamic biocompatibility indices. This paradigm shift will accelerate the translation of i-TE technology from laboratory demonstrations to practical clinical tools.
